# Multivariate linear regression analysis to identify general factors for quantitative predictions of implant stability quotient values

**DOI:** 10.1371/journal.pone.0187010

**Published:** 2017-10-30

**Authors:** Hairong Huang, Zanzan Xu, Xianhong Shao, Daniel Wismeijer, Ping Sun, Jingxiao Wang, Gang Wu

**Affiliations:** 1 Department of Oral Implantology and Prosthetic Dentistry, Academic Centre for Dentistry Amsterdam (ACTA), Universiteit van Amsterdam and Vrije Universiteit Amsterdam, Amsterdam, Nord-Holland, the Netherlands; 2 Department of Prosthodontic Dentistry, Johann Wolfgang Goethe University, Frankfurt, Hessen, Germany; 3 Best & Easy Dental Clinic, Hangzhou, Zhejiang Province, P.R. China; 4 The Affiliated Stomatology Hospital, Zhejiang University School of Medicine, Hangzhou, Zhejiang Province, P.R. China; 5 The First Affiliated Hospital, Wenzhou Medical University, Wenzhou, Zhejiang Province, P.R. China; University of Memphis, UNITED STATES

## Abstract

**Objectives:**

This study identified potential general influencing factors for a mathematical prediction of implant stability quotient (ISQ) values in clinical practice.

**Methods:**

We collected the ISQ values of 557 implants from 2 different brands (SICace and Osstem) placed by 2 surgeons in 336 patients. Surgeon 1 placed 329 SICace implants, and surgeon 2 placed 113 SICace implants and 115 Osstem implants. ISQ measurements were taken at T1 (immediately after implant placement) and T2 (before dental restoration). A multivariate linear regression model was used to analyze the influence of the following 11 candidate factors for stability prediction: sex, age, maxillary/mandibular location, bone type, immediate/delayed implantation, bone grafting, insertion torque, I-stage or II-stage healing pattern, implant diameter, implant length and T1-T2 time interval.

**Results:**

The need for bone grafting as a predictor significantly influenced ISQ values in all three groups at T1 (weight coefficients ranging from -4 to -5). In contrast, implant diameter consistently influenced the ISQ values in all three groups at T2 (weight coefficients ranging from 3.4 to 4.2). Other factors, such as sex, age, I/II-stage implantation and bone type, did not significantly influence ISQ values at T2, and implant length did not significantly influence ISQ values at T1 or T2.

**Conclusions:**

These findings provide a rational basis for mathematical models to quantitatively predict the ISQ values of implants in clinical practice.

## Introduction

Dental implantation has become one of the most widely used treatment options for partially or completely edentulous patients in the past several decades. Dental implants serve as artificial roots in jaw bones without the risk of damaging natural teeth and mechanically support various upper dentures, such as crowns, bridges and overdentures. The mechanical stability of implants forms the biological basis for implant functions. A sufficient primary stability must be achieved immediately after implantation via the mechanical engraving of the implant into the surrounding bone, which provides an indispensable mechanical microenvironment for the gradual establishment of secondary stability. Primary stability plays a dominant role in implant stability in the first week after implantation and decreases significantly thereafter to a minimal level at approximately 5 weeks [1]. Secondary stability is based on the biological process osseointegration, during which a direct structural contact between the implant surfaces and the new surrounding bone tissues is formed [2]. Secondary stability increases after implantation and rapidly increases from 2.5 weeks to a plateau level 5 or 6 weeks after implantation. The entire process of transition from primary to secondary stability takes approximately 5–8 weeks [1]. Implant stability is used as a major indicator in clinical practice to determine the time frame for loading and prognosis of the implants (failure) [3]. Many methods, such as resonance frequency analysis (RFA), have been developed to estimate implant stability.

RFA has become one of the most widely used techniques to assess implant stability in clinical practice [4]. RFA is performed by measuring the response of an implant-attached piezo-ceramic element to a vibration stimulus consisting of small sinusoidal signals in the range of 5 to 15 kHz in steps of 25 Hz on the other element. The peak amplitude of the response is encoded into a parameter called the implant stability quotient (ISQ), which ranges from 0 to 100 [5]. The ISQ value reflects positively the general mechanical stability of an implant. A more precise prediction of ISQ values would help surgeons determine the possible loading scheme for the patient and assess the long-term survival probability of dental implants [4]. However, various clinical factors influence ISQ values, and many clinical trials investigated the influence of different clinical factors on ISQ measuring results. However, most clinical trials focused on one or several parameters only, which may help only in qualitative assessments of the influence of various factors on future ISQ measurements but which are unable to quantitatively predict ISQ values (and mechanical stability) during the healing course. In our recent study, we used a new model by performing a multivariate linear regression analysis to filter out and quantify the most significant contributions of selected factors from 11 candidate factors to ISQ values during the healing course of an implant [6]. In this study, we analyzed the data of 329 patients receiving SICace implants treated by one surgeon (group 1). ISQ values at T1 and T2 were influenced by implant diameter and the insertion torque. The ISQ values obtained at T1 were influenced specifically by the sex of the patient, location (maxillary or mandibular), implantation mode (immediate/delayed implantation), healing stage (time factor) and the absence or presence of bone graft material. Other factors also played a role, such as implant design, including the macrodesign (thread design and body shape) microdesign (implant topography) [5], the drilling technique [7], and the preparation technique of the surgical site [8]. We hypothesized that an equation may be related specifically to the surgeon and the implant system used in clinical practice. We formulated the following two hypotheses based on these findings: First, the key factors influencing the ISQ values are dependent on the dental implant type used, the surgeon and his/her techniques; and second, surgeon- and implant system-independent general factors influence these key factors. If one factor significantly influenced the ISQ values consistently in the three groups at T1 or T2, then we categorized that factor as a general influencing factor. It is of paramount significance for the surgeon to identify the potential general influencing factors that are applicable for other surgeons and other implant systems. We collected data on SICace implants from one surgeon and data of SICace implants and Osstem implants from another surgeon to identify the potential general factors that consistently and significantly (or insignificantly) influenced ISQ values.

## Materials and methods

### Patients and implants

The conduct of this study was approved by the Review Boards of the Best & Easy Dental Clinic and Huayang Dental Clinic, People’s Republic of China. It is routine for all patients at both dental clinics to provide an informed written consent for potential inclusion in clinical studies. In this retrospective study, the data of 329 SICace implants (SIC Invent AG, Basel, Switzerland) from surgeon no. 1 were obtained from BEST&DENTAL Clinic, Hangzhou, China, from 2012 to 2015 (group 1) as we reported earlier [6]. We also reviewed the data of all the patients who received implant treatment in the Huayang Dental Clinic, Cixi, China, from 2012 to 2015; and we also included 113 SICace implants (SIC Invent AG, Basel, Switzerland) from 81 patients (group 2) and 115 TSIII implants (OSSTEM, Seoul, Korea) from 78 patients treated by surgeon no. 2 (group 3). There was 1 implant failure in 114 SIC (the failure rate was 0.9%), and there were 2 implant failures in 117 TSIII (the failure rate was 1.7%) over this time period. The data of the 3 failed implants were not included in the subsequent analysis.

### General inclusion and exclusion criteria for implant treatments

In both dental clinics, we adopted the patients for implant treatment based on the same grounds and criteria: if they were classified as ASA1, ASA2 and ASA3, according to the American Society of Anesthesiology (ASA) classifications. Patients with uncontrolled or severe periodontitis were excluded, as well as pregnant patients.

### Patient records

We retrospectively reviewed the following parameters from the patients: (X1) sex; (X2) age; (X3) maxillar/mandibular location; (X4) immediate/delayed implantation; (X5) presence or absence of bone grafting; (X6) implant diameter; (X7) implant length; (X8) I/II-stage healing pattern; (X9) insertion torque; (X10) bone type; and (X11) T1-T2 time interval (see S1 File). The II-stage healing method was used only if the insertion torque was <20 Ncm or the ISQ value was <65. The bone type of the implantation sites were categorized into types I, II, III and IV, according to the classification of Lekholm and Zarb [9].

ISQ values were measured using an Osstell™ Mentor (Integration Diagnostic Ltd., Goteborg, Sweden) from the mesial, distal, lingual and buccal sites of each implant at T1 (measured immediately at the time of implant placement) and T2 (measured before dental restoration). The mean ISQ values from the four sites were used for statistical analyses.

### Statistical analysis

We performed multivariate linear regression analyses to determine the weight coefficients of the 11 candidate factors at the T1 and T2 time points. All statistical analyses were performed using SPSS® 21.0 software (SPSS, Chicago, IL, USA). The level of significance was set at *p<*0.05, and the confidence level was set at 95%. We also performed an unpaired t test to compare the results with the model we established. The following influencing factors were transformed into numerical values: (X1) male = 1, female = 2; (X3) maxillary = 1, mandible = 2; (X4) immediate = 1, delayed = 2; (X5) bone grafting: no = 1, yes = 2, and (X8) I-stage = 2, II-stage = 1. Dummy variables were used for bone types (X10): type 1 = 100, type 2 = 010, type 3 = 001, and type 4 = 000. The statistical analyses for the data in group 1, 2 and 3 respectively can be found in S2 File.

## Results

**Fig 1:**
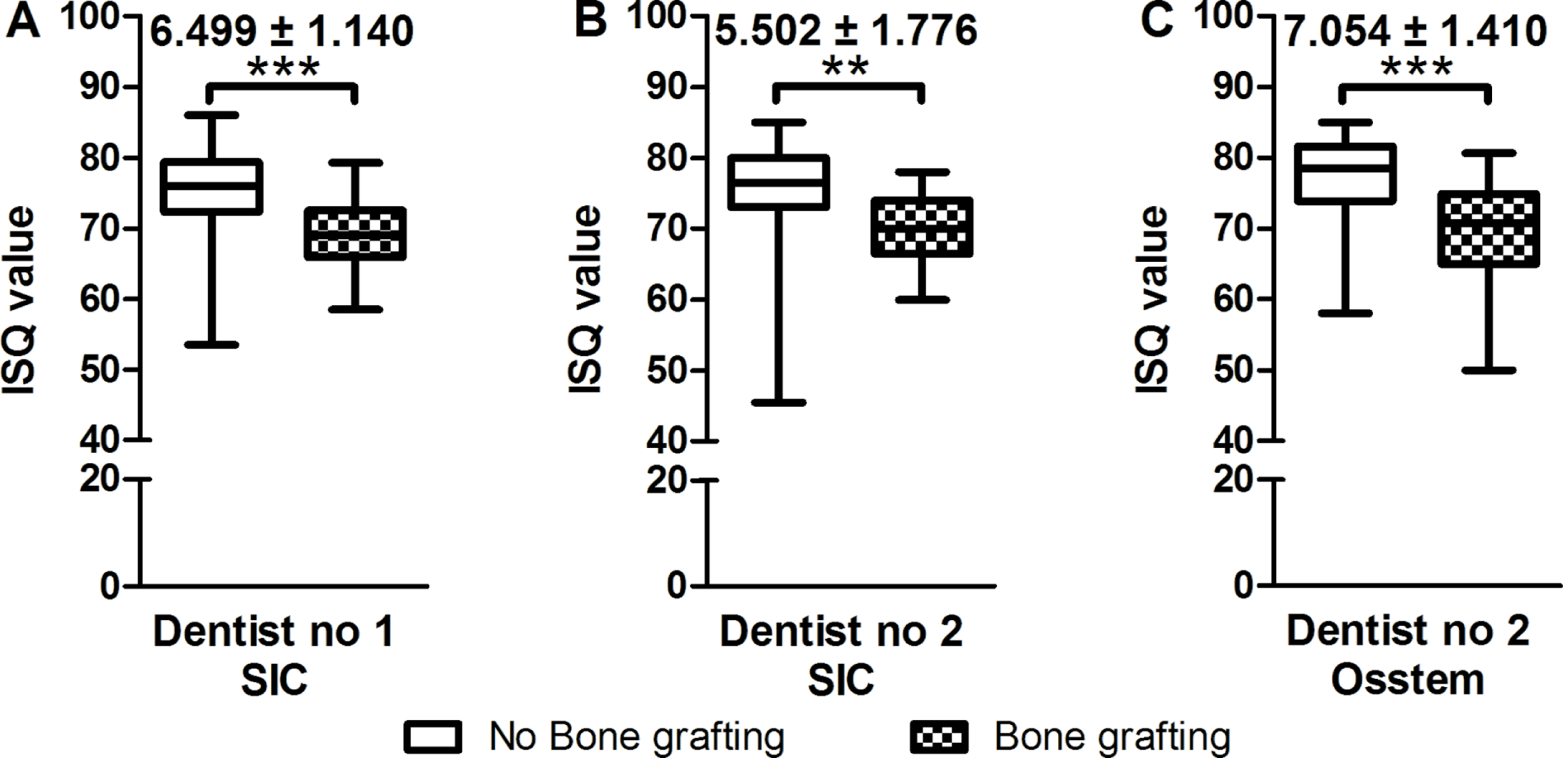
The influence of bone grafting on the values of Implant Stability Quotients (ISQ). n: implant numbers. Data are presented as means with min and max. ^&^: The differences of mean values between the two corresponding groups.

Table 1 lists the descriptive characteristics of all the patients and implants. The need for bone grafting (X5) significantly influenced ISQ values in all three groups at T1 (immediately after implantation), and the non-standardized coefficients ranged from -4 to -5 (Table 2). Unpaired t test demonstrated that X5 was a significant influencing factor for all three groups, and the influence of X5 (from -5.5 to -7.1) was larger than the range estimated by our model (Fig 1). In contrast, X7 (implant length) did not significantly influence ISQ values at T1 or T2. X6 (implant diameter) consistently influenced ISQ values in all three groups at T2, with coefficients ranging from -3.4 to -4.2 (Table 3). In contrast, sex (X1), age (X2), I/II-stage implantation (X8) and bone type (X10) did not significantly influence ISQ values at T2 (Table 3).

**Table 1:** Descriptive characteristics of patients and implants.

Characteristics and Factors (X)		Category	Group 1 Surgeon no. 1 SICace	Group 2 Surgeon no. 2 SICace	Group 3 Surgeon no. 2 Osstem
	Number of patients		177	81	78
	Number of implants		329	113	115
X1	Sex	Male	103	36	33
		Female	74	45	45
X2	Age (years)	19–30	18	15	12
		31–40	65	24	16
		41–50	70	25	27
		51–60	86	35	32
		61–70	50	13	23
		71–80	25	1	5
		81–100	5	0	0
		Missing data	10	0	0
X3	Maxillary/mandible location	Maxilla	112	40	55
		Mandibular	217	73	60
X4	Immediate/delayed implantation	Immediate	103	44	25
		Delayed	226	69	90
X5	The need of bone graft	Yes	27	24	36
		No	302	89	79
X6	Implant diameter (mm)	3.5	30	18	0
		3.7	0	0	19
		4	203	89	0
		4.2	0	0	27
		4.5	58	0	59
		5	38	6	10
X7	Implant length (mm)	7.5	6	6	0
		8.5	0	0	22
		9.5	120	52	0
		10	0	0	56
		11.5	103	34	18
		13	95	20	19
		14.5	5	1	0
X8	I/II-stage healing pattern	I-stage	105	89	73
		II-stage	224	24	42
X9	Insertion torque (Ncm)	10–20	38	17	22
		21–30	99	38	26
		31–40	52	42	60
		41–50	118	14	7
		51–60	7	2	0
		missing data	15	0	0
X10	Bone type	1	95	21	13
		2	51	69	67
		3	62	15	17
		4	83	8	18
		missing data	38	0	0
X11	T1-T2 time interval (months)	1.5	21	2	1
		2	30	2	0
		2.5	37	0	0
		3	25	0	0
		3.5	47	0	0
		4	30	51	66
		5	31	30	16
		6–9	81	28	32
		missing data	27	0	0

**Table 2:** Multivariate linear regression analyses of the weight coefficients of each influencing factor for the values of Implant Stability Quotients (ISQ) that were measured immediately after implantation T1.

Constant and Influencing factors (X)	Unstand. Coef. β±SE		
	Group 1 Surgeon no. 1 SICace	Group 2 Surgeon no. 2 SICace	Group 3 Surgeon no. 2 Osstem
Constant	57.263±4.226***	57.444±4.470***	62.730±3.556***
X1	1.317±.622*	─	─
X2	─	0.143±0.051**	─
X3	1.471±.652*	─	─
X4	1.836±.664**	─	─
X5&	-4.990±1.135***	-4.006±1.638*	-4.117±1.255***
X6	1.669±.754*	─	─
X7§	─	─	─
X8	2.961±.657***	─	4.948±1.234***
X9	0.131±.025***	─	0.277±0.069***
X10 (1, 2, 3)	─	7.590±3.119*	─

Unstand. Coef.: Unstandardized Coefficients. (X1): Sex; (X2): Age; (X3): Maxillary/mandibular location; (X4): Immediate/delayed implantation; (X5): the need for Bone grafting; (X6): Implant diameter; (X7): Implant length; (X8): I/II-stage implantation; (X9): Insertion torque; (X10) Bone type; and (X11): T1-T2 time interval.

& indicates significant general influencing factors.

§ indicates insignificant general influencing factors.

*: 0.01<P≤0.05

**: 0.001<P≤0.01

***: P≤0.001.

**Table 3:** Multivariate linear regression analyses of the weight coefficient of each influencing factor for the values of Implant Stability Quotient (ISQ) measured just prior to loading T2.

Constant and Influencing factors (X)	Unstand. Coef. β±SE		
	Group 1 Surgeon no. 1 SICace	Group 2 Surgeon no. 2 SICace	Group 3 Surgeon no. 2 Osstem
Constant	56.988±3.043***	73.198±7.275***	50.608±4.765***
X1§	─	─	─
X2§	─	─	─
X3	─	─	2.646±0.752***
X4	─	─	4.628±1.002***
X5	─	-2.665±1.111*	─
X6&	4.080±0.698***	3.454±1.222**	4.197±1.194***
X7§	─	─	─
X8§	─	─	─
X9	0.048±0.698*	─	─
X10§	─	─	─
X11	0.014±0.005**	─	─

Unstand. Coef.: Unstandardized Coefficients. (X1): Sex; (X2): Age; (X3): Maxillary/mandibular location; (X4): Immediate/delayed implantation; (X5): the need of Bone grafting; (X6): Implant diameter; (X7): Implant length; (X8): I/II-stage implantation; (X9): Insertion torque; (X10) Bone type; and (X11): T1-T2 time interval.

& indicates significant general influencing factors.

§ indicates insignificant general influencing factors.

*: 0.01<P≤0.05

**: 0.001<P≤0.01

***: P≤0.001.

## Discussion

ISQ values are frequently used and highly important in clinical practice to estimate implant stability and assess prognosis. A more precise prediction of ISQ values will allow surgeons to take appropriate measures at earlier time points during the implant healing course and reduce the risk of failures. However, most previous analyses only provide a coarse qualitative evaluation of the significance and role of one or several influencing factors. A shortage of useful and practical methodologies to precisely, mathematically and more accurately predict the ISQ values of implants remains. In our recent study, we formulated a mathematical model to estimate the weight coefficients of candidate factors for a more precise assessment of primary and secondary implant stabilities [6]. The primary goal of this model is to provide a practical tool for surgeons to predict the ISQ values of patient implants and plan early and appropriate corrective therapeutic measures. This type of model may be personalized to the surgeon and his/her methods and be specific to implant types. Whether this model may be used to analyze the general influencing factors of (future) ISQ values is not known. Therefore, we created the current model to analyze the data of one implant type from two different surgeons and the data of two types of implant systems (from the same surgeon) in this study. We found that the need for bone grafting (X5) and implant diameter (X6) were the most significant influencing factors, irrespective of the surgeon or implant type, for future ISQ values at T1 and T2, respectively.

The need for bone grafting (X5) was the only significant general influencing factor at T1 (Table 2). We attributed this finding to the fact that the bone coverage of the implants would be significantly smaller if bone grafting was needed. Notably, the weight coefficients of the three groups ranged from -4 to -5, which are quite close to each other. This finding suggests that surgeons may conclude that bone grafting will result in ISQ values less than 4 to 5, which is precisely the clinical significance that our study aimed to provide as a practical and calculable method to predict ISQ values. The use of conventional analyses with unpaired t tests to evaluate the influence of X5 on ISQ values also produced a significant difference between groups with and without bone grafting (Fig 1). However, these difference values ranged from -5.5 to -7.1, which is much larger than the values obtained in our model. This difference may be attributed to the fact that the influence of other factors was not considered in the conventional method and remained unbalanced. This factor became even less pronounced or non-significant in influencing ISQ values at T2 (Table 2), which made it a generally non-significant influencing factor.

Several in vitro studies previously demonstrated that longer implants were associated with significantly higher ISQ values than shorter implants [10,11]. Two recent publications found that the correlative relationship of implant length and ISQ values was restricted to implants with diameters of 3.8 mm [12]. Bataineh and Al-Dakes [13] demonstrated that only an implant length of 15 mm significantly correlated to ISQ values. However, clinical studies did not confirm these findings, and these correlations may occur in special cases. In contrast, these results demonstrated that implant length did not significantly influence primary stability results [14]. Our data demonstrated that implant length (X7) did not significantly influence ISQ values (in all the three groups) at T1 or T2 time points, which is consistent with these clinical findings. This finding suggests that attempts to increase primary and secondary stability using longer implants in clinical practice has no solid scientific base.

Implant diameter is another implant design-related factor that may influence implant stabilities. A recent in vitro biomechanical study using insertion torque as a parameter found that wider implants were associated with significantly higher insertion torques in hard bone than narrower implants [15]. However, these differences were not significant for primary stability values because no significant differences in ISQ values were found. These phenomena may be attributed to a much smaller correlation between micromotion and insertion torque values than ISQ measurements [16]. Han et al. [17] demonstrated that ISQ values did not correlate with implant diameter values over a 12-week post-operative monitoring time period in a small-scale prospective clinical trial. Several studies also demonstrated that implant diameters did significantly influence ISQ values [18,19]. In our current study, we found that implant diameter was a general significant influencing factor only, at T2. This finding is consistent with our previous report that found the influence of implant diameter on ISQ values was much larger at T2 than T1. Notably, the coefficients were 4.080±0.698, 3.454±1.222 and 4.197±1.194 for the three groups of implants, which are quite similar values. This finding suggested the ability to quantitatively predict ISQs at T2; the 1.5-mm-diameter difference between the 3.5-mm and 5-mm implants could be transformed into a difference of 5.175 to 6.296 (calculated by multiplying 1.5 by 3.454 and 4.197) in ISQ values.

Primary implant stability is related to the immediate mechanical engagement of an implant with the surrounding bone, which is established at the time of implant insertion [13]. Secondary stability depends on bone formation and remodeling at the implant-bone interface over time, and it is influenced by the geometrical, chemical and biological properties of the implant surface and wound-healing time [20–22]. Some researchers examined whether bone-to-implant contact (BIC) correlated to the implant stability quotient. However, the degree of osseointegration did not correlate with ISQ values, particularly when only the BIC values were measured, (i.e., the bone-implant–contact percentage) [23,24]. BIC is only a relative bone-coverage value of the implant surface area, but it ignores the anchoring bony trabeculae that are needed to establish the structural connectivity of the implant surface with the parent bone surface. Hagi et al. [25] recently discussed these aspects.

We also found several general insignificant influencing factors at T2, such as sex (X1), age (X2), I/II-stage implantation (X8) and bone type (X10). The influence of sex on implant stability was variable and inconsistent in previous reports. Males exhibited significantly higher [26], significantly lower [27] or similar [28] ISQ values compared to females. In our study, sex showed no significant influence in 2 of the 3 groups at T1 and in none of the groups at T2, which suggests a minimal importance of sex in predicting ISQ values. The influence of age as a general factor exhibited a similar pattern.

Bone type was not a significant influencing factor on implant stability previously [14], and baseline microstructural bone characteristics that were assessed using histomorphometric and microtomographic analyses revealed no significant influence on implant stability [29]. Bone type was only important in one group at T1 in our study, which exhibited a rather high weight coefficient of 7.590±3.119. Whether this result may be attributed to the relatively low number of type-4 bone cases in this group is not clear. The availability of a larger sample size may provide additional data for clarification of this aspect. Another possible reason for bone type is that the classification is very roughly categorized. A recent study [30] of bone typing using only semi-quantitative data for CBCT and bone types demonstrated that the identification of the bone type itself remains completely subjective. Factor X8 (I/II-stage implantation) significantly influenced ISQ values at T1 in two of our 3 groups with high weight coefficients (2.961±.657 and 4.948±1.234). These influences exhibited a surgeon-specific or implant type-dependent characteristic. These influences were absent at T2. However, a previous study demonstrated that a I/II-stage implantation did not result in different degrees of osseointegration [31]. Further investigations should be performed to clarify the influencing pattern of I/II-stage implantation when surgeons wish to obtain predictive information on ISQ values.

Another interesting coincidence occurred for maxillary/mandibular location (X3) and immediate/delayed implantation (X4). Both factors significantly influenced SICace implants from surgeon 1 at T1 and Osstem implants from surgeon 2 at T2. These influences were moderate at T1 and robust at T2 and clearly not negligible. However, the limits of this study prevented any correlation of these findings to a rational pattern.

One clear limitation of this study was the limitations in the setup of the groups. We only had two groups for the same surgeon or same implant system. Furthermore, the numbers of implants were not completely comparable in the three groups, which may have influenced the power of the statistical analyses. Therefore, careful interpretation is needed if extrapolations are planned based on the current data to estimate ISQ values for other implant types. However, the results of the current study should encourage surgeons to undertake multivariate linear regression analyses and establish their own equations. A growing accumulation of these equations will establish more precise evidence-based models to predict ISQ values in clinical practice.

## Supporting information

S1 File Database of three groups. Excel file to record all the collected data of the included patients.
(XLSX)

S2 File Statistical analyses. Statistical analyses for the data in group 1, 2 and 3 respectively.
(DOCX)
