# Supplementary material for: Multivariate linear regression analysis to identify general factors for quantitative predictions of implant stability quotient values
Source: PLoS One. 2017 Oct 30;12(10):e0187010. doi: 10.1371/journal.pone.0187010 (PMC5662232; doi:10.1371/journal.pone.0187010)
Supplement: S2 File — Statistical analyses for the data in group 1, 2 and 3 respectively. (DOCX) [file pone.0187010.s002.docx]

Statistics report for group 1

SPSS 21.0 analysis, $\alpha$=0.05,average+SD（$\bar{x}\pm s$）, if the samples meet the requirement of normality and homogeneity of variance between groups using analysis of variance，if not use Kruskal-Wallis；use multivariate linear regression analyses to find out the key factors influence ISQ1 and ISQ2，dummy variables were used for bone types，if the samples don’t meet the normality and homogeneity of variance, eliminate the variables to satisfy the requirement of the model establishment.

Result

1. Comparison of ISQ value measured at distal, mesial, buccal and lingual direction

Table 1 normality and homogeneity of variance test

| group | | Kolmogorov-Smirnov(K) | | |
| --- | --- | --- | --- | --- |
|  |  | Statistic |  | Sig. |
| ISQ1 | M | 0.097 |  | 0.000 |
|  | D | 0.110 |  | 0.009 |
|  | B | 0.098 |  | 0.000 |
|  | L | 0.106 |  | 0.000 |
| ISQ2 | M | 0.072 |  | 0.026 |
|  | D | 0.085 |  | 0.114 |
|  | B | 0.102 |  | 0.000 |
|  | L | 0.112 |  | 0.000 |

，

Kruskal-Wallis test

Table2 comparison of ISQ at distal ,mesial ,buccal and lingual direstion

| Group | M | D | B | L | $\chi^{2}$ | P |
| --- | --- | --- | --- | --- | --- | --- |
| ISQ1 | 74.85±6.486 | 74.09±6.652 | 74.02±7.193 | 74.40±6.865 | 2.810 | 1.781 |
| ISQ2 | 77.26±4.781 | 76.65±4.751 | 76.97±5.048 | 77.14±4.988 | 0.442 | 0.619 |

1. Multivariate linear regression analyses

ISQ 1:

Y1=57.263+0.131 X9--4.990 X5+2.961 X8+1.471X3+1.836X4+1.669X6+1.317X1

Table 3 results of multivariate linear regression

| model | Unstandardized Coefficients | | Standardized  Coefficients Beta | t | Sig. | 95.0% Confidence Interval for B | |
| --- | --- | --- | --- | --- | --- | --- | --- |
|  | B | Std. Error |  |  |  | Lower Bound | Upper Bound |
| (Constant) | 57.263 | 4.226 | - | 13.551 | .000 | 48.942 | 65.585 |
| X9 | .131 | .025 | .286 | 5.313 | .000 | .082 | .180 |
| X5 | -4.990 | 1.135 | -.235 | -4.395 | .000 | -7.226 | -2.754 |
| X8 | 2.961 | .657 | .241 | 4.504 | .000 | 1.666 | 4.255 |
| X3 | 1.471 | .652 | .121 | 2.257 | .025 | .188 | 2.755 |
| X4 | 1.836 | .664 | .148 | 2.763 | .006 | .527 | 3.144 |
| X6 | 1.669 | .754 | .119 | 2.212 | .028 | .183 | 3.154 |
| X1 | 1.317 | .622 | .111 | 2.116 | .035 | .091 | 2.542 |

ISQ 2:

Y2=56.988+4.080X6+0.014X11+0.048X9

Table 4. results of multivariate linear regression

| model | Unstandardized Coefficients | | Standardized Coefficients  Beta | t | Sig. | 95.0% Confidence Interval for B | |
| --- | --- | --- | --- | --- | --- | --- | --- |
|  | B | Std. Error |  |  |  | Lower Bound | Upper Bound |
| (Constant) | 56.988 | 3.043 | - | 18.726 | .000 | 50.977 | 63.000 |
| X6 | 4.080 | .698 | .414 | 5.848 | .000 | 2.702 | 5.459 |
| X11 | .014 | .005 | .191 | 2.715 | .007 | .004 | .025 |
| X9 | .048 | .023 | .150 | 2.115 | .036 | .003 | .093 |

Statistics report for group 2

SPSS 21.0 analysis, $\alpha$=0.05,average+SD（$\bar{x}\pm s$）, if the samples meet the requirement of normality and homogeneity of variance between groups using analysis of variance，if not use Kruskal-Wallis；use multivariate linear regression analyses to find out the key factors influence ISQ1 and ISQ2，dummy variables were used for bone types，if the samples don’t meet the normality and homogeneity of variance, eliminate the variables to satisfy the requirement of the model establishment.

**Result**

1. Comparison of ISQ value measured at distal, mesial, buccal and lingual direction

Table 1 normality and homogeneity of variance test

| groups | | Kolmogorov-Smirnov(K) | | |
| --- | --- | --- | --- | --- |
|  |  | Statistic |  | Sig. |
| ISQ1 | m | 0.130 |  | 0.001 |
|  | d | 0.111 |  | 0.189 |
|  | b | 0.116 |  | 0.004 |
|  | l | 0.110 |  | 0.033 |
| ISQ2 | m | 0.204 |  | <0.001 |
|  | d | 0.196 |  | <0.001 |
|  | b | 0.158 |  | <0.001 |
|  | l | 0.186 |  | <0.001 |

Kruskal-Wallis test

Table2 comparison of ISQ at distal ,mesial ,buccal and lingual direstion

| Group | m | d | b | l | H | Q |
| --- | --- | --- | --- | --- | --- | --- |
| ISQ1 | 74.17±7.684 | 73.51±6.785 | 73.46±7.401 | 73.13±7.449 | 1.380 | 0.710 |
| ISQ2 | 78.00±4.514 | 77.04±4.398 | 76.57±5.156 | 76.45±5.272 | 3.177 | 0.365 |

1. Multivariate linear regression analyses

ISQ 1:

Y1=57.444+0.143X2+2.500X3+0.114X9-4.006X5+7.985X10(1)+7.552X10(2)+7.324X10(3)

| model | Unstandardized Coefficients | | Standardized  Coefficients Beta | t | Sig. | 95.0% Confidence Interval for B | |
| --- | --- | --- | --- | --- | --- | --- | --- |
|  | B | Std. Error |  |  |  | Lower Bound | Upper Bound |
| (Constant) | 57.444 | 4.470 |  | 12.851 | .000 | 48.550 | 66.338 |
| X2 | .143 | .051 | .259 | 2.785 | .007 | .041 | .245 |
| X3 | 2.500 | 1.431 | .170 | 1.747 | .084 | -.347 | 5.347 |
| X9 | .114 | .063 | .185 | 1.808 | .074 | -.011 | .240 |
| X5 | -4.006 | 1.638 | -.229 | -2.446 | .017 | -7.265 | -.748 |
| bone1 | 7.895 | 3.086 | .421 | 2.559 | .012 | 1.755 | 14.034 |
| bone2 | 7.552 | 2.940 | .529 | 2.569 | .012 | 1.703 | 13.401 |
| bone3 | 7.324 | 3.331 | .401 | 2.199 | .031 | .696 | 13.951 |

ISQ2

Y2=73.198-0.606X7+3.454X6-2.665X5

Table 4 results of multivariate linear regression

| model | Unstandardized Coefficients | | Standardized Coefficients  Beta | t | Sig. | 95.0% Confidence Interval for B | |
| --- | --- | --- | --- | --- | --- | --- | --- |
|  | B | Std. Error |  |  |  | Lower Bound | Upper Bound |
| (Constant) | 73.198 | 7.275 |  | 10.062 | .000 | 58.733 | 87.662 |
| X7 | -.606 | .337 | -.207 | -1.799 | .076 | -1.275 | .064 |
| X6 | 3.454 | 1.222 | .305 | 2.826 | .006 | 1.024 | 5.884 |
| X5 | -2.665 | 1.111 | -.235 | -2.400 | .019 | -4.873 | -.457 |

Statistics report for group 3

SPSS 21.0 analysis, $\alpha$=0.05,average+SD（$\bar{x}\pm s$）, if the samples meet the requirement of normality and homogeneity of variance between groups using analysis of variance，if not use Kruskal-Wallis；use multivariate linear regression analyses to find out the key factors influence ISQ1 and ISQ2，dummy variables were used for bone types，if the samples don’t meet the normality and homogeneity of variance, eliminate the variables to satisfy the requirement of the model establishment.

1. Comparison of ISQ value measured at distal, mesial, buccal and lingual direction

Table 1 normality and homogeneity of variance test

| group | | Kolmogorov-Smirnov(K) | | |
| --- | --- | --- | --- | --- |
|  |  | Statistic |  | Sig. |
| ISQ1 | M | 0.160 |  | <0.001 |
|  | D | 0.143 |  | <0.001 |
|  | B | 0.131 |  | <0.001 |
|  | L | 0.113 |  | 0.004 |
| ISQ2 | M | 0.156 |  | <0.001 |
|  | D | 0.164 |  | <0.001 |
|  | B | 0.144 |  | <0.001 |
|  | L | 0.158 |  | <0.001 |

Kruskal-Wallis test

Table2 comparison of ISQ at distal ,mesial ,buccal and lingual direstion

| Time point | M | D | B | L | H | P |
| --- | --- | --- | --- | --- | --- | --- |
| T1 | 74.72±7.751 | 74.20±7.740 | 74.78±7.137 | 75.03±7.151 | 0.365 | 0.947 |
| T2 | 81.54±5.114 | 81.39±5.258 | 80.77±5.328 | 80.95±5.157 | 2.034 | 0.565 |

1. multivariate linear regression analyses for group 3

ISQ 1:

Y1=62.730+0.277X9+4.948X8-4.117X5

Table 3 results of multivariate linear regression

| model | Unstandardized Coefficients | | Standardized  Coefficients Beta | t | Sig. | 95.0% Confidence Interval for B | |
| --- | --- | --- | --- | --- | --- | --- | --- |
|  | B | Std. Error |  |  |  | Lower Bound | Upper Bound |
| (Constant) | 62.730 | 3.556 | - | 17.641 | <0.001 | 55.673 | 69.788 |
| X9 | 0.277 | 0.069 | 0.328 | 4.013 | <0.001 | 0.140 | 0.414 |
| X8 | 4.948 | 1.234 | 0.326 | 4.008 | <0.001 | 2.498 | 7.397 |
| X5 | -4.117 | 1.255 | -.0262 | -3.279 | .001 | -6.609 | -1.625 |

ISQ2

Y2=50.608+4.628X4+2.646X3+4.197X6

Table 4 results of multivariate linear regression

| model | Unstandardized Coefficients | | Standardized Coefficients  Beta | t | Sig. | 95.0% Confidence Interval for B | |
| --- | --- | --- | --- | --- | --- | --- | --- |
|  | B | Std. Error |  |  |  | Lower Bound | Upper Bound |
| (Constant) | 50.608 | 4.765 | - | 10.621 | .000 | 41.151 | 60.065 |
| X4 | 4.628 | 1.002 | .387 | 4.618 | .000 | 2.639 | 6.616 |
| X3 | 2.646 | .752 | .268 | 3.519 | .001 | 1.154 | 4.139 |
| X6 | 4.197 | 1.194 | .292 | 3.514 | .001 | 1.827 | 6.568 |
